# Retrotransposon Silencing by DNA Methylation Can Drive Mammalian Genomic Imprinting

**DOI:** 10.1371/journal.pgen.0030055

**Published:** 2007-04-13

**Authors:** Shunsuke Suzuki, Ryuichi Ono, Takanori Narita, Andrew J Pask, Geoffrey Shaw, Changshan Wang, Takashi Kohda, Amber E Alsop, Jennifer A. Marshall Graves, Yuji Kohara, Fumitoshi Ishino, Marilyn B Renfree, Tomoko Kaneko-Ishino

**Affiliations:** 1 Department of Epigenetics, Medical Research Institute, Tokyo Medical and Dental University, Tokyo, Japan; 2 Genome Biology Laboratory, National Institute of Genetics, Mishima, Shizuoka, Japan; 3 Department of Zoology, University of Melbourne, Victoria, Australia; 4 Research School of Biological Sciences, the Australian National University, Canberra, Australia; 5 School of Health Sciences, Tokai University, Bohseidai, Isehara, Kanagawa, Japan; University of Cambridge, United Kingdom

## Abstract

Among mammals, only eutherians and marsupials are viviparous and have genomic imprinting that leads to parent-of-origin-specific differential gene expression. We used comparative analysis to investigate the origin of genomic imprinting in mammals. *PEG10* (paternally expressed 10) is a retrotransposon-derived imprinted gene that has an essential role for the formation of the placenta of the mouse. Here, we show that an orthologue of *PEG10* exists in another therian mammal, the marsupial tammar wallaby *(Macropus eugenii),* but not in a prototherian mammal, the egg-laying platypus *(Ornithorhynchus anatinus),* suggesting its close relationship to the origin of placentation in therian mammals. We have discovered a hitherto missing link of the imprinting mechanism between eutherians and marsupials because tammar *PEG10* is the first example of a differentially methylated region (DMR) associated with genomic imprinting in marsupials. Surprisingly, the marsupial DMR was strictly limited to the 5′ region of *PEG10,* unlike the eutherian DMR, which covers the promoter regions of both *PEG10* and the adjacent imprinted gene *SGCE*. These results not only demonstrate a common origin of the DMR-associated imprinting mechanism in therian mammals but provide the first demonstration that DMR-associated genomic imprinting in eutherians can originate from the repression of exogenous DNA sequences and/or retrotransposons by DNA methylation.

## Author Summary

Genomic imprinting is a gene regulatory mechanism controlling parent-of-origin-dependent expression of genes. In eutherians, imprinting is essential for fetal and placental development and defects in this mechanism are the cause of several genetic disorders. In eutherian mammals, genomic imprinting is controlled by differential methylation of the DNA. However, no such methylation-dependent mechanism had been previously identified in association with marsupial imprinting. By comparing the genome of all three extant classes of mammals (eutherians, marsupials, and monotremes), we have investigated the evolution of *PEG10* (paternally expressed 10), a retrotransposon-derived imprinted gene that is essential for the formation of the placenta in the mouse. *PEG10* was present in a marsupial species, the tammar wallaby, but absent from an egg-laying monotreme species, the platypus. Therefore, *PEG10* was inserted into the genome at the time when the placenta and viviparity were evolving in therian mammals. This study has shown that *PEG10* is not only imprinted in a marsupial, but that its imprint is regulated by differential methylation, suggesting a common origin for methylation in the therian ancestor. These results provide direct evidence that retrotransposon insertion can drive the evolution of genomic imprinting in mammals.

## Introduction

Genomic imprinting, or parent-of-origin-specific gene silencing, has been observed in both eutherian and marsupial, but not monotreme mammals. In eutherians, more than 80 imprinted genes have been found and differential DNA methylation plays a crucial role in regulating their imprinted expression patterns [1–5]. In marsupials, three genes—*IGF2*, *IGF2R,* and *PEG1/MEST*—are imprinted, as they are in eutherians, but no differentially methylated regions (DMRs) have been found in marsupials [6–8]. Consequently, the regulatory mechanisms of genomic imprinting were thought to have evolved differently between marsupials and eutherians [6,8,9].

It has been hypothesized that genomic imprinting arose as a by-product of a DNA methylation mechanism that silences foreign DNAs [10], such as retrotransposons [11,12]. Similarly, transgenes can also become methylated, depending on parent of origin, further supporting a link between genomic imprinting and silencing of foreign DNAs [13–15]. *PEG10* is an imprinted gene sharing homology with the sushi-ichi retrotransposon, and in humans and mice it has a clear DMR in its promoter region. Interestingly, *PEG10* is conserved in eutherian mammals but not in nonmammalian vertebrates, such as birds and fish [11,16–18]. Therefore, investigating the origin of the retrotransposon-derived *PEG10* locus would clarify the relationship between retrotransposon (or exogenous DNA sequence) insertion and genomic imprinting.

*PEG10* is an essential placental gene in eutherians, since knock-out mice have severe placental defects with loss of spongiotrophoblast and labyrinth layers leading to early embryonic lethality [18]. The origin of *PEG10* is therefore of interest in view of its possible contribution to the evolution of mammalian placentation. Sequence identified as *PEG10* has recently been reported in the South American marsupial the grey short-tailed opossum (Monodelphis domestica) [16] but its precise location, genetic structure, and imprint status remains unknown. Here, we examined the *PEG10* locus in two Australian mammals by isolating bacterial artificial chromosome (BAC) clones from a marsupial, the tammar wallaby, and from a monotreme, the platypus.

## Results/Discussion

**Figure 1:**
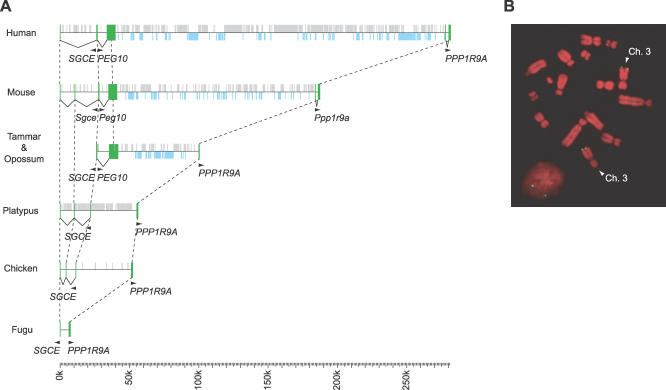
Comparison of the Genomic sStructures between *SGCE* and *PPP1R9A,* and Chromosomal Location for Tammar *PEG10* (A) The green bars on the horizontal lines indicate the exons of each gene. Orthologous exons are connected between species by the broken lines. The grey bars on the upper side of the horizontal lines represent LINEs and SINEs, and the blue bars on the underside represent LTR elements. The arrowheads indicate the direction of transcription. The published opossum genome sequence used was *PEG10* downstream, and was combined with our tammar genome sequence at the homologous point in the 3′ UTR of *PEG10*. (B) Fluorescence in situ hybridization of *PEG10* was localized to Chromosome 3q. There is a nucleus of another cell in the lower left.

We used the tammar wallaby and the platypus as the representative species of marsupials and monotremes, respectively, to compare with representative eutherians, the human and the mouse. Several BAC clones were isolated from tammar and platypus containing *SGCE* (sarcoglycan epsilon), the neighboring gene of *PEG10* in eutherians. DNA sequencing of one tammar BAC clone demonstrated the existence of *PEG10* and its conserved location adjacent to *SGCE* with transcription occurring in a head-to-head manner (Figure 1A). The genetic structure of tammar *PEG10* was also the same as that of eutherians, with two open reading frames (ORFs) related to the sushi-ichi retrotransposon GAG and POL proteins, respectively [11]. The CX_2_CX_4_HX_4_C RNA-binding motif of GAG and the DSG sequences of the proteinase activation site of POL were also conserved (Figure S1). Tammar *PEG10* was localised close to the telomere of Chromosome 3q, consistent with its autosomal location on proximal mouse Chromosome 6 (Figure 1B). However, in the platypus, there were no *PEG10* homologous sequences between *SGCE* and *PPP1R9A* (also called *NEURABIN1*) that flank *PEG10* in other mammals (Figure 1A).

Using our tammar sequence and the published opossum genome sequence, we compared the entire region between *SGCE* and *PPP1R9A* with the equivalent region in several vertebrates from fish (fugu) to mammals. The size of this region in the platypus was similar to that of the chicken and was smaller than in other mammals. There were numerous long interspersed nuclear elements (LINEs) and short interspersed nuclear elements (SINEs) (grey bars in Figure 1A) in all mammalian groups. A previous report suggests that there are less long terminal repeat (LTR)-type retrotransposon-derived sequences in the opossum and wallaby genomes [19], but a large number of these sequences were found in this region as well as in mouse and human (blue bars in Figure 1A). Consistent with the previous report, these were absent in the platypus [19], as was *PEG10*. Most of the LTR-type retrotransposon-derived sequences observed in these regions are species specific. This suggests that most of these insertions occurred after species diversification. The presence of *PEG10* sharing homology with one of the LTR-type retrotransposons, sushi-ichi [11], in both marsupials and eutherians suggests that the original *PEG10* sequence insertion in the common therian ancestor was an early event in the therian-specific expansion of LTR-type retrotransposons. These results indicate that the original *PEG10* was inserted into the genome of the therian ancestor and evolved to its present function as an essential placental gene after divergence from the monotremes. The acquisition of a new function for an existing character during evolution, a process termed “exaptation” by Gould and colleagues, would be enhanced by the provision of novel genetic materials, such as retrotransposons [20,21]. Thus, the requirement for *PEG10* in placental function is a clear example of “exaptation.” The fossil record shows that there was extensive radiation of therian mammals after their split from the Prototheria. New LTR-type retrotranposon-derived sequences might therefore have contributed novel genetic resources to this radiation.

**Figure 2:**
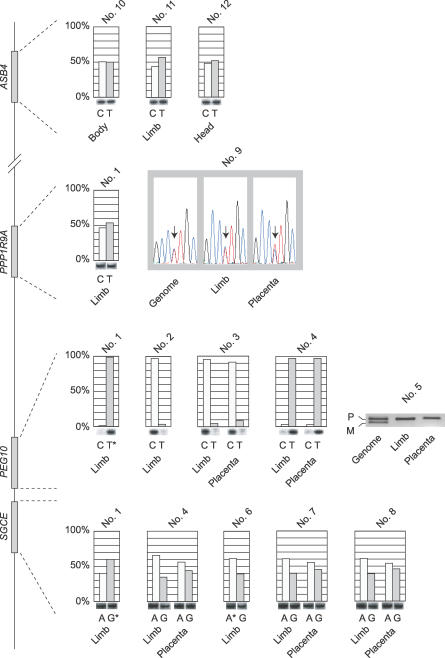
Allelic Expression Analysis of Tammar *PEG10* and Neighboring Genes The individual animal numbers are shown above the graphs and the pictures. The pairs of white and grey bars in the graphs represent the two parental alleles in each individual. The vertical axes represent the expression ratios of each allele percentage of total expression, and the raw data are shown under each bar. The characters under the raw data indicate the residues at each polymorphic site, and asterisks are added to the paternal alleles in the informative cases. The gel picture for No. 5 shows the result of RT-PCR for the individual with a *PEG10* length polymorphism. Parental origin of both alleles are represented by “P” for paternal and “M” for maternal, respectively. The picture for No. 9 shows the results of direct sequencing for each PCR product. The arrows indicate the polymorphic sites.

In mice, there is a large imprinting cluster near *Peg10* which includes the paternally expressed *Sgce* gene [22,23] *Ppp1r9a,* which is maternally biased in extraembryonic tissues [23] and *Asb4,* which is completely maternally expressed in both embryos and the extraembryonic tissues [23,24]. Tammar *PEG10* showed almost complete monoallelic expression in all individuals. Paternal expression was confirmed in two embryos and one yolk sac placenta sample (Figure 2). Unexpectedly, tammar *SGCE* showed predominantly biallelic expression with only a small paternal bias, despite a short 200-bp distance between the transcription start sites of *PEG10* and *SGCE* (Figure 2). *PPP1R9A* and *ASB4* showed biallelic expression without parental bias (Figure 2). These results clearly demonstrate that imprinting in this region is restricted to the *PEG10* gene in the tammar. As described above, the eutherian *PEG10* imprinted region includes several neighboring genes, suggesting that the imprinted region expanded in the eutherians while in marsupials imprinting was restricted to *PEG10.*

**Figure 3:**
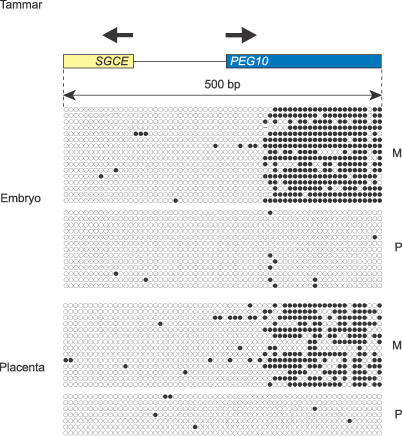
DNA Methylation Status of the CpG Island over the Promoter Regions of *SGCE* and *PEG10* The regions corresponding to *SGCE* and *PEG10* are represented by the boxes. *SGCE* (yellow box) has biallelic expression whilst *PEG10* (blue box) shows paternal expression. Black circles indicate methylated CpGs and white circles indicate unmethylated CpGs. Parental origin of each clone is distinguished by the polymorphism and the mother's genotype, and is represented by “P” for paternal and “M” for maternal, respectively.

A CpG island is present in the promoter regions of *SGCE* and *PEG10* in tammar as well as in mouse. To determine why *SGCE* did not show imprinted expression we examined its methylation status. Surprisingly, we found a DMR with a clear boundary of DNA methylation between the *PEG10* side and the *SGCE* side of the CpG island in both embryos and yolk sac placentas (Figure 3). Furthermore, selective DNA methylation of the maternal allele was confirmed using a DNA polymorphism in this region as predicted by the paternal expression of *PEG10.* The DNA methylation started about 60 bp downstream from the transcription start site of *PEG10,* suggesting that maternal transcription is inhibited by methylation of downstream regulatory elements and not by the typical mechanism of promoter methylation (Figure 3). Both LTR [25] and non-LTR retrotransposons [26] are known to have internal transcriptional regulatory elements for their transcription. As *PEG10* is a retrotransposon-derived gene, these elements may exist within the DMR not, as is usual, upstream of the transcription start site. A part of the CpG island was possibly derived from LTRs in the original *PEG10* sequence and the methylation that originated from a host–defense mechanism may be restricted to the ancient repetitive-element homologous region. Alternatively, a boundary function for DNA methylation spreading and/or transcription regulation may be included in the marsupial CpG island. The retrotransposon LTR sequences are CpG-rich and have such a boundary function in Saccharomyces cerevisiae and Drosophila melanogaster [27,28]. In *Drosophila*, the boundary function has been attributed to the binding of the SU(HW) protein. A consensus SU(HW) binding site was not found around the methylation boundary in the tammar *PEG10*. However, the CTCF protein, well known to have a similar insulator/boundary function in mammals, may bind to the possible boundary elements containing CT-rich sequences in this region.

**Figure 4:**
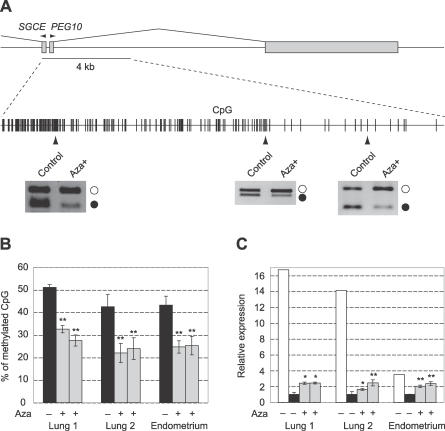
DNA Methylation and Allelic Expression of *PEG10* in 5-Aza-2′-deoxycytidine Treated Tammar Cells (A) The bars on the horizontal line represent each CpG site. Three CpG sites were examined for DNA methylation status using combined bisulphite restriction analysis indicated by the arrowheads. The intensity of the bands marked with the white and black circles show the amount of unmethylated and methylated CpG, respectively. (B) Repetitive experiments and quantification of the cut and uncut bands of combined bisulphite restriction analysis were performed for the most 3′ site indicated in Figure 4A. One black and two grey bars represent the results of positive control cells and of two independent 5-aza-2′-deoxycytidine treated cells, respectively. The decrease in methylation was statistically significant (**) (*p* < 0.01). Quantification of each samples was performed three times using independent PCR products. (C) Relative expression was calculated by quantifying the results of restriction fragment length polymorphism analysis. White and black bars represent the expression from active and inactive alleles of positive control cells, respectively. It should be noted that expression from the inactive alleles was negligible. Two grey bars represent induced expression from the inactive alleles of two independent 5-aza-2′-deoxycytidine treated cells. Statistically significant increase in expression after treatment is shown by * (*p* < 0.03) or ** (*p* < 0.01).

Even with the presence of a DMR, it is possible that the maternal copy of *PEG10* in the tammar is silenced by another mechanism and is only secondarily methylated. We therefore examined whether the imprinted expression of tammar *PEG10* was regulated by DNA methylation. A reduced level of DNA methylation was observed in three sites of the CpG island in cells cultured with 5-aza-2′-deoxycytidine, a DNA methylation inhibitor (Figure 4A). Repetitive experiments performed for the most 3′ site using three independent cell lines established from fetal lung and endometrium also showed statistically significant reductions in DNA methylation levels (Figure 4B), and increased *PEG10* expression from normally repressed alleles was observed in each case (Figure 4C, black and grey bars), although the expression levels were still much lower than active alleles (Figure 4C, white bars). These results demonstrate the association between imprinted expression of *PEG10* and DNA methylation in a marsupial, although it still remains unknown if the differential methylation originates in the germline as does a typical primary DMR in eutherians.

The DNA methylation status of retrotransposons can differ between male and female germ cells. For example, IAP and LINE1 are more highly methylated in sperm than oocytes, while *Alu* is less methylated [12]. Mice and humans with paternal disomy that express *PEG10* and *SGCE* biallelically have normal phenotypes, so monoallelic expression of these genes is not essential for development. Therefore, although *PEG10* is essential for placental development in the mouse, the imprinting of this locus may be a functionally unimportant inheritance derived from the nature of the original retrotransposition of *PEG10*.

**Figure 5:**
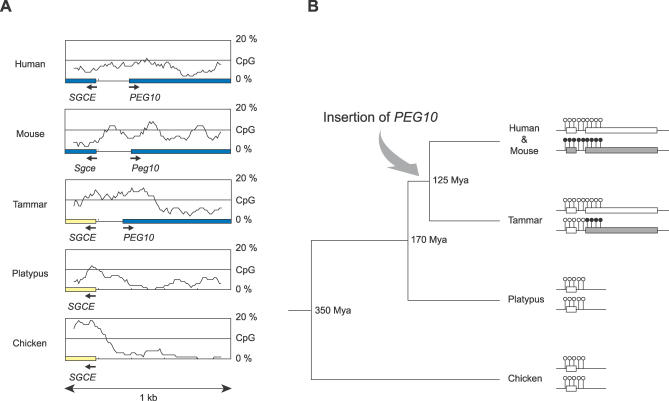
Comparisons of the Genomic Region Surrounding *SGCE* in Various Species and Phylogeny of *PEG10* Imprinting (A) CpG content of a 1-kb genomic sequence starting in the conserved end of the exon1 in *SGCE* is shown in each species. The regions corresponding to *SGCE* and *PEG10* are represented by boxes below. Yellow boxes indicate biallelic expression and blue boxes represent paternal expression. Precise start sites of *SGCE* in chicken and platypus remain unknown. (B) Illustration of the insertion of the original *PEG10* sequence in therian not prototherian lineage at least 125 million years ago [31,32]. The short and long boxes represent *SGCE* and *PEG10,* respectively. The black lollipops indicate DNA methylation of CpG sites and the grey boxes represent the silent state of transcription.

There were CpG islands in the putative promoter region of *SGCE* of the chicken, platypus, tammar, mouse, and human (Figure 5A). We hypothesize that insertion of *PEG10* after the divergence of therian from prototherian mammals expanded the CpG islands (Figure 5). In the tammar, DNA methylation is restricted to *PEG10*, but in the mouse and human, the entire region is differentially methylated. These differences may be explained by the presence or absence of a boundary function of the CpG island in these groups as discussed above. However, in both cases, insertion of *PEG10,* which must have occurred in the therian ancestor, is clearly sufficient to establish imprinting of this region (Figure 5B). Our study confirms that silencing of exogenous DNA after retrotransposon insertion can drive the evolution of genomic imprinting in mammals.

## Materials and Methods

### Animals and tissue collection.

Tammar wallabies of Kangaroo Island origin were maintained in our breeding colony in grassy, outdoor enclosures. Lucerne cubes, grass and water were provided ad libitum and supplemented with fresh vegetables. Fetuses and yolk sac placenta tissue were collected between days 22 and 25 of the 26.5-d gestation [29]. Experimental procedures conformed to Australian National Health and Medical Research Council (1990) guidelines and were approved by the Animal Experimentation Ethics Committees of the University of Melbourne.

### Isolation of BAC clones and determination of genomic DNA sequences.

Each BAC clone in the tammar and platypus BAC libraries was stored separately and was spotted onto nylon membranes correspondently. These membranes were hybridized with the partial *SGCE* probes of each species, and the positive clones were identified according to the locus information of the signals on the membranes. The tammar *PEG10* sequence was determined by the primer walking method from a partial fragment amplified using cross-species degenerate primers. The platypus sequence between *SGCE* and *PPP1R9A* was determined by the shotgun sequencing method, and it was completed using a published database of whole genome shotgun sequences and direct sequencing of PCR products.

### Detection of repetitive sequences.

RepeatMasker (http://www.repeatmasker.org) was used for the detection of LINEs, SINEs, and LTR elements in the genomic region between *SGCE* and *PPP1R9A*.

### Fluorescence in situ hybridization.

BAC DNA was labeled by nick translation with digoxygenin-11-dUTP. Hybridization was operated with the labeled BAC DNA and C_0_t-1 DNA at 37 °C overnight. Anti-digoxygenin-Cy3 and DAPI were used for the detection of the signals and for the counterstain, respectively.

### Single nucleotide primer extension assay.

The details have been described in our previously published paper [8]. The 3′ UTRs of *PEG10* and *ASB4* including the polymorphisms were amplified by 30–35 cycles of RT-PCR using the following pairs of primers: *PEG10*-F1, 5′-CAAATGCCATTGCCGTCT-3′ and *PEG10*-R1, 5′-GTTAGACGGTCAGCTCCACG-3′; *PEG10*-F2, 5′-CAACCAGGGGAGCTAGGATT-3′ and *PEG10*-R2, 5′-GAACATCCATGCACCGTAGA-3′; *PEG10*-F3, 5′-CTCTCTGGAGCGGTATCCAG-3′ and *PEG10*-R3, 5′-TGTGAGATTTGGCAATCATACA-3′; and *ASB4*-F, 5′-AACACCCCGAGGTCTCTCAT-3′ and *ASB4*-R, 5′-GAGGACCATGGCATTTATTCA-3′. The following correspondent SNuPE primers were used for the single nucleotide extension: *PEG10*-SN1, 5′-ATTCATTTCCCTTCCCAACAT-3′; *PEG10*-SN2, 5′-CCCTGGCTGCGAGACCA-3′; *PEG10*-SN3, 5′-CCCGGGGAGCTCCGAGC-3′; and *ASB4*-SN, 5′-CAAGAAGCAAGTAGTTCTTCAAAGG-3′

### Hot stop PCR.

The labeling of the primers for the last hot cycle was operated using [γ-^32^P]ATP and T4 DNA kinase. The final PCR products of *SGCE* and *PPP1R9A* were digested by Alul and Mspl, respectively. The 3′ UTRs of *SGCE* and *PPP1R9A* including the polymorphisms on the recognition sequences of these restriction enzymes were amplified by 30–35 cycles of PCR using the following pairs of primers. Asterisks indicate the labeled primers: *SGCE*-F, 5′-CAGTGATGGCGTTCTGTACG-3′; *SGCE-R, 5′-GTTGATGACCAGGTTGTGCC-3′; *PPP1R9A-F, 5′-CCAGGAGAAGATGGAGAAGC-3′; and *PPP1R9A*-R, 5′-GTTGGGGATGAAGGAGTGTG-3′. Ten percent polyacrylamide gels were used for the gel electrophoresis of the digested samples.

### Bisulphite sequencing.

After the bisulphite treatment [30] for the genomic DNA of tammar, the region corresponding to the CpG islands over the promoter regions of *SGCE* and *PEG10* was amplified by 35 cycles of PCR using the following pair of primers: CGI-F, 5′-GGAGTGATTGTGGAAATGGAGGTG-3′ and CGI-R, 5′-ATACAAAATCCCCCCCCTAAACCTC-3′. The PCR products were cloned and the clones were analyzed by sequencing.

### Cell culture.

Primary culture of tammar fetal lung cells from day 25 of gestation and adult endometrium cells were used in this study. Control cells were cultured in 50% AmnioMAX (Invitrogen, http://www.invitrogen.com) and 50% DMEM supplemented with 10% fetal calf serum and penicillin/streptomycin at 37 °C/5% CO_2_. Cells for 5-aza-2′-deoxycytidine treatment were cultured in the same media but containing 10 μM of 5-aza-2′-deoxycytidine (Sigma). Fresh media with 5-aza-2′-deoxycytidine were added every 24 h for 6 d.

### Combined bisulphite restriction analysis.

Three regions in the *PEG10* DMR were amplified by 35 (for the middle and right side in Figure 4A) and 40 (for the left side in Figure 4A) cycles of PCR from the bisulphite treated genomic DNA of cells using the following pair of primers: LEFT-F, 5′-GTATTAGTTTTTTTGTAGTT-3′ and LEFT-R, 5′-CCTAAAAAACTACCCTACTCC-3′; MID-F, 5′- GAGATGGGGAGATTGATATTT-3′ and MID-R, 5′- CCCTATAACTAAACTACAATCTCTCC-3′; and RIGHT-F, 5′- CCTCCCATTAACTTTAAAATCACC-3′ and RIGHT-R, 5′- ATTGTAGTAATGGGGTAGGTTATG-3′.

PCR products were digested by RsaI (for the left side) or Aci I (for the middle and right side) for analyses.

### Restriction fragment length polymorphism analysis.

The 3′ UTR of *PEG10,* including the polymorphism on the TaqI or BceAI recognition sequences, was amplified by 30 cycles of RT-PCR using the *PEG10*-F1R1 or *PEG10*-F3R3 primer pairs. PCR products were digested by TaqI or BceAI for analyses.

## Supporting Information

Figure S1 Amino Acid Sequence Alignment between Human and Tammar PEG10 Amino acid sequence identity (asterisks), homology (dots), and divergence (no marks) between human and tammar PEG10 (ORF1 and 2 are combined) are shown. The red boxes represent the conserved CCHC and DSG motifs.
(15 KB PDF)

### Accession Numbers

The National Center for Biotechnology Information GenBank (http://www.ncbi.nlm.nih.gov/Genbank) sequence accession numbers for tammar and platypus BACs are AB260975 and AB260976, respectively.

## Abbreviations

BAC: bacterial artificial chromosome
DMR: differentially methylated region
LINES: long interspersed nuclear elements
LTR: long terminal repeat
SINES: short interspersed nuclear elements
