# Supplementary material for: Retrotransposon Silencing by DNA Methylation Can Drive Mammalian Genomic Imprinting
Source: PLoS Genet. 2007 Apr 13;3(4):e55. doi: 10.1371/journal.pgen.0030055 (PMC1851980; doi:10.1371/journal.pgen.0030055)
Supplement: Supplementary file 1 [file pgen.0030055.sg001.pdf]

Fig. S1

```
Tammar PEG10    1' MPTSTGSGASARRCRMEDLTAEVQALRAQLLQQTEENSSLQTQLLQLSEENANLRGQLQA
                                 *...*..*...**.....*.***..**.*. .*.***..**.*...
Human PEG10     1"           MTERRRDELSEEINNLREKVMKQSEENNNLQSQVQKLTEENTTLREQVEP

Tammar PEG10   61' TP------------VGAMGLSAP-RGKCPVGLPEKFDGTPEMLPSFLAQSRLYMELRPED
                   **            ..* . ..*  ..** .*******.*.**..*.**....** .. *
Human PEG10    51" TPEDEDDDIELRGAAAAAAPPPPIEEECPEDLPEKFDGNPDMLAPFMAQCQIFMEKSTRD

Tammar PEG10  108' FPTEHVRVCFLTSLLKGRAARWATPYLLESSPLLNNYEAFIDEFKQAFEDPQRKEAANRK
                   *....*****.**...*******.. *  *  *..** **. *.*..******.*.*.**
Human PEG10   111" FSVDRVRVCFVTSMMTGRAARWASAKLERSHYLMHNYPAFMMEMKHVFEDPQRREVAKRK

Tammar PEG10  168' IRRLRQGLGSVLDYASAFRLCAQDLDWNEPAFIDQFLEGLSDPIQDELARVEVARSLPAL
                   *******.***.**..**.. **********.***. *****.**.**...***.**.**
Human PEG10   171" IRRLRQGMGSVIDYSNAFQMIAQDLDWNEPALIDQYHEGLSDHIQEELSHLEVAKSLSAL

Tammar PEG10  228' INQCLRIEGRL----NSRRGASPASS-PQPQSASSPPVESESNQLVTRPQLTPEERERRR
                   *.**..** **    ..*.  **...   *. ** ..*....    .* .**.**.****
Human PEG10   231" IGQCIHIERRLARAAAARKPRSPPRALVLPHIASHHQVDPTEPVGGARMRLTQEEKERRR

Tammar PEG10  283' RLNLCMYCGLAGHYVDNCPSKKPKSSGVGNSLAPLGKLPGPAVEGPPAPGPEPERSPPDD
                   .****.*** .***.****.* .*** .*** **************.*.***  ***.**
Human PEG10   291" KLNLCLYCGTGGHYADNCPAKASKSSPAGNSPAPLGKLPGPAVEGPSATGPEIIRSPQDD

Tammar PEG10  343' ASSSHLQLLITLHLPDGSYCTVRAMIDSGASGNFIDQDYVAQTGIPVKIKYWPVLVEAID
                   ***.***... .***.     ***************..****.***..** **.******
Human PEG10   351" ASSPHLQVMLQIHLPGRHTLFVRAMIDSGASGNFIDHEYVAQNGIPLRIKDWPILVEAID

Tammar PEG10  403' GRPLASGPVTQETQELVVDIGEHRERLSFDVTQSPFFPVVLGVRWLTQHNPSIMWSSRTI
                   ***.*****..**..*.**.*.*** ********************. *.*.* **.*.*
Human PEG10   411" GRPIASGPVVHETHDLIVDLGDHREVLSFDVTQSPFFPVVLGVRWLSTHDPNITWSTRSI

Tammar PEG10  463' VFDSEYCQHNCRAALLMAPSLVPGGMD--------------------VQEVLVPMAQEAI
                   *******...**    ..*** * . .                    **.* .*.....
Human PEG10   471" VFDSEYCRYHCRMYSPIPPSLPPPAPQPPLYYPVDGYRVYQPVRYYYVQNVYTPVDEHVY

Tammar PEG10  503' P--------YHAPYSYTVAPTGYPYAVSDPELMAVRDFLAKNVKWVCVQPA-SPLGAPVV
                   *         .   .    *.*. *..*.**. *.***.*.***   . *. .* **.*.
Human PEG10   531" PDHRLVDPHIEMIPGAHSIPSGHVYSLSEPEMAALRDFVARNVKDGLITPTIAPNGAQVL

Tammar PEG10  554' IMKKVGELYLCNDSQNPNAIAVWNQYPPPSTPYV--LYQLQAAPIYRTRSPAGYTTWSML
                    .*.  .* .. *...**.... ****. *.* .    .*... . ..  * ** *..
Human PEG10   591" QVKRGWKLQVSYDCRAPNNFTIQNQYPRLSIPNLEDQAHLATYTEFVPQIP-GYQTYPTY

Tammar PEG10  612' GREMNGKLCLIPFLVTSNIR

Human PEG10   650" AAYPTYPVGFAWYPVGRDGQGRSLYVPVMITWNPHWYRQPPVPQYPPPQPPPPPPPPPPP
```
